# Criticality-Enhanced Magnetocaloric Effect in Quantum Spin Chain Material Copper Nitrate

**DOI:** 10.1038/srep44643

**Published:** 2017-03-15

**Authors:** Jun-Sen Xiang, Cong Chen, Wei Li, Xian-Lei Sheng, Na Su, Zhao-Hua Cheng, Qiang Chen, Zi-Yu Chen

**Affiliations:** 1 Department of Physics, Key Laboratory of Micro-Nano Measurement-Manipulation and Physics (Ministry of Education), Beihang University, Beijing 100191, China; 2 International Research Institute of Multidisciplinary Science, Beihang University, Beijing 100191, China; 3 Department of Physics and Astronomy, University of Delaware, Newark, Delaware 19716-2570, USA; 4 State Key Laboratory of Magnetism and Beijing National Laboratory for Condensed Matter Physics, Institute of Physics, Chinese Academy of Sciences, Beijing 100190, China

## Abstract

In this work, a systematic study of Cu(NO_3_)_2_·2.5 H_2_O (copper nitrate hemipentahydrate, CN), an alternating Heisenberg antiferromagnetic chain model material, is performed with multi-technique approach including thermal tensor network (TTN) simulations, first-principles calculations, as well as magnetization measurements. Employing a cutting-edge TTN method developed in the present work, we verify the couplings *J* = 5.13 K, *α* = 0.23(1) and Landé factors *g*_∥_= 2.31, *g*_⊥_ = 2.14 in CN, with which the magnetothermal properties have been fitted strikingly well. Based on first-principles calculations, we reveal explicitly the spin chain scenario in CN by displaying the calculated electron density distributions, from which the distinct superexchange paths are visualized. On top of that, we investigated the magnetocaloric effect (MCE) in CN by calculating its isentropes and magnetic Grüneisen parameter. Prominent quantum criticality-enhanced MCE was uncovered near both critical fields of intermediate strengths as 2.87 and 4.08 T, respectively. We propose that CN is potentially a very promising quantum critical coolant.

Heisenberg spin chains and nets, owing to their strong quantum fluctuations and correlation effects, can accommodate plentiful interesting quantum phases like topological spin liquids^1^,^2^, unconventional excitations like anyon-type quasi particles^3^, and inspiring behaviors like Bose- Einstein condensation in magnets^4^, which continues stimulating both condensed matter theorists and experimentalists. What is more, these low-dimensional systems, which at a first glance are of purely academic interest, can actually have their experimental realizations. People have successfully discovered and synthesized plenty of spin materials which are very well described by the low-dimensional Heisenberg-type spin models. The long list includes, to name only a few, the diamond spin chain material azurite^5^, the kagome spin liquid herbertsmithite^6^, and Cu(NO_3_)_2_·2.5 H_2_O (copper nitrate hemipentahydrate, hereinafter referred to as “CN”) as an alternating Heisenberg antiferromagnetic chain (AHAFC)^7^,^8^,^9^,^10^,^11^,^12^,^13^,^14^,^15^,^16^,^17^,^18^,^19^,^20^,^21^,^22^,^23^,^24^,^25^,^26^,^27^,^28^,^29^.

Among many other interesting properties of low-dimensional quantum magnets, we emphasize the enhanced magnetocaloric effect (MCE) in quantum critical regime. MCE is an intrinsic property of magnetic materials which exploits the reversible entropy changes caused by varying magnetic fields. MCE has a long history of study^30^,^31^,^32^, and in the past decades, developing novel MCE materials which have prominent MCE properties, like the Gadolinium alloys with giant MCE^33^,^34^, has raised great research interest. This is due to that MCE has appealing applications in eco-friendly refrigeration near room temperature^33^,^35^, which provides a good substitute to conventional vapor compression refrigeration, and also can be utilized in space technology^36^,^37^. In addition, MCE materials, in particular adiabatic dimagnetization refrigerant (ADR), serve as efficient coolants for realizing ultra low temperatures^38^,^39^,^40^,^41^. People pursues MCE refrigerant which have higher isothermal entropy change (Δ*S*), larger adiabatic temperature difference (*T*_ad_), and also lower hysteresis dissipation^34^.

Recently, quantum spin chain materials are shown to exhibit enhanced MCE even at ultra low temperatures, and thus raised great research interest^40^,^41^,^42^,^43^,^44^,^45^,^46^,^47^,^48^,^49^. On one hand, through exploring low-*T* MCE properties of spin chain model materials^41^,^42^ which shows divergent Grüneisen parameter near field-induced quantum critical points (QCPs), people are able to directly detect and study quantum criticality^43^,^44^. On the other hand, one can inversely utilize this low-temperature thermodynamic anomaly to realize enhanced cooling effects near QCPs^46^,^47^. Very recently, Sharples *et al*. realized temperatures as low as ∼200 mK using the enhanced MCE of a molecular quantum magnet^40^, and Lang *et al*. experimentally studied a spin-1/2 Heisenberg antiferromagnetic chain material [Cu(*μ*-C_2_O_4_)(4-aminopyridine)_2_(H_2_O)]_*n*_ (CuP, for short)^48^, and demonstrated this quantum critical coolant is a perfect alternative to standard ADR salts, due to its wider operating temperature range, longer holding time and higher efficiency^49^. As a typical low-dimensional quantum spin chain material, magnetic refrigeration of CN has also been experimentally explored, but only under a magnetic field range far from the field-induced QCPs^8^.

In order to study the thermodynamic information including the appealing MCE property of these strongly correlated spin systems, accurate thermal algorithms are of crucial significance, which is indispensable in establishing links between theoretical spin models and experimental measurements at finite temperatures. In one spatial dimension (1D), the transfer matrix renormalization group (TMRG) method^50^,^51^,^52^ has been long accepted as the method of reference, owing to its high accuracy and versatility. In ref. 53, Li *et al*. proposed an alternative approach for calculating thermodynamics of low-dimensional quantum lattice models called linearized tensor renormalization group (LTRG) method, which also adopts the Trotter-Suzuki decomposition^54^ to express the partition function as a *d* + 1 (*d* = 12 for 1D and 2D lattices, respectively) dimensional thermal tensor network (TTN) and linearly contract it along Trotter direction via renormalization group (RG) techniques.

In this work, combining three different methods, i.e., thermal quantum manybody computations, *ab initio* calculations, and experimental measurements of magnetization, we performed a comprehensive investigation of an AHAFC material CN. It is one of the earliest inorganic spin chain material ever studied experimentally^7^,^8^,^9^,^10^,^11^,^12^,^13^,^14^,^15^,^16^,^17^,^18^,^19^,^20^,^21^,^22^,^23^,^24^,^25^,^26^,^27^,^28^,^29^,^55^, while continues intriguing people for its abundant physics including triplon wave excitation^23^ and precise Tomanaga-Lutting liquid behavior^29^. We notice that, despite many efforts, discrepancy in coupling constants still exists: the exact diagonalization (ED) fittings (*J* = 5.16 K, *α* = 0.27) to thermodynamic quantities measurably deviates from those obtained from inelastic neutron scattering (INS) experiments (*J* = 5.14 K, *α* = 0.227)^26^.

We hereby utilize the LTRG approach with a bilayer formulation (dubbed as LTRG++) which further improves the accuracy of calculations^56^. With this cutting-edge TTN method at hand, we revisit the previous experimental data in ref. 22 including specific heat curves (at various fields) and magnetization curves, augmented with magnetization measurements done by us. The couplings are verified to be precisely *J* = 5.13 K, *α* = 0.23(1), consistent with that from INS experiments. In addition, first-principles calculations present electron density distributions and therefore visualized superexchange paths, thus providing direct and indubitable proof on the spin-chain alignment in material CN. Furthermore, through TTN simulations, we show that CN has large entropy change and pronounced peaks (and dips) in Grüneisen parameter around QCPs at low temperatures, and the calculated adiabatic temperature changes can fit very well to the previously measured isentropes, revealing that CN may be an ideal quantum critical refrigerant.

## Results

### Alternating Heisenberg antiferromagnetic spin chain material copper nitrate

As one of the common copper salts, CN possesses some special thermodynamic properties at low temperatures (see in Supplementary Note 1), including the zero-magnetization plateau^11^,^20^, 1D Luttinger liquid behavior under magnetic fields^29^,^57^, and 3D magnetic transition at ultra low temperature (150∼160 mK)^16^,^19^,^29^, etc, which has been arousing people’s research interest for more than half a century, significantly promoting developments of the research on low-dimensional quantum magnets.

**Figure 1:**
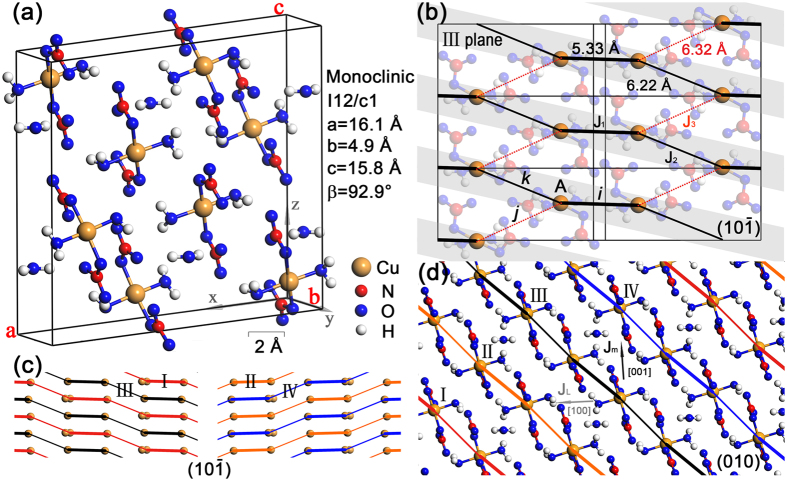
Crystal structure and magnetic exchange couplings in Cu(NO_3_)_2_·2.5 H_2_O. (**a**) The unit cell of Cu(NO_3_)_2_·2.5 H_2_O, where the coordinate axes coincide with the crystal axes. The lattice constants are shown in the figure, indicating that CN belongs to the monoclinic system. (**b**) Structure in a typical (10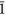) plane, where the Cu^2+^ are highlighted while other atoms left transparent. The heavy solid lines are the intradimer *J*_1_, the interdimer *J*_2_ and interchain *J*_3_ couplings are plotted differently (in black solid and red dashed lines, respectively). *A* labels one out of two sublattices of honeycomb lattice in (10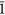) plane, and 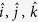 are vectors connecting one site (in *A* sublattice) with its three nearest neighbors. (**c**) Superexchange paths between spins along chains in four inequivalent (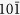) planes which are adjacent to each other. (**d**) Projected view of the crystal structure in (010) plane, where the alternating solid lines represent the *J*_1_–*J*_2_ chains. We denote the four existing (10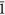) planes as I, II, III, and IV, respectively, where the chains have different paths in each plane. Arrows indicate the directions along [100] and [001], which represent interchain exchange paths *J*_L_, *J*_m_.

Figure 1 depicts the crystallographic structure of CN, which is monoclinic with space group *I*12/*c*1^9^. The spin chain structure and the spin-spin interaction paths can be seen in Fig. 1(b–d). The distances between one Cu^2+^ to its three neighbors are 5.33 Å, 6.22 Å, and 6.32 Å^18^, which leads to three distinct couplings *J*_1_, *J*_2_, and *J*_3_, respectively [Fig. 1(b)]. We depict two possible inter-dimer superexchange paths *J*_2_ and *J*_3_ in Supplement Fig. S2, and Fig. 1(b) shows that the spin chain could have had two possible routes on 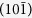 planes. Until recently, INS determines that *J*_3_ = −0.01 meV (of magnitude about 1/10 of *J*_2_)^28^, so that *J*_2_ is confirmed to be the dominant inter-dimer interaction, which connects dimers to form a tilted alternating chain, as shown in Fig. 1(b–d).

Therefore, it is concluded that an AHAFC model can very well describes the magnetic properties of CN (in the temperature regime above ∼160 mK), which reads


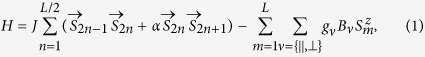


where 
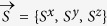
 is the vector spin operators in different directions; *J* = *J*_1_ is the strongest superexchange coupling; *α* = *J*_2_/*J*_1_ is the relative strength of dominant inter-dimer interaction, whose precise value was measurably different in various experiments and left undetermined between 0.227 and 0.27^22^,^26^. Also note that in the magnetic-field coupling (Zeeman) term, the Landé factors are different (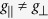) on the direction along *b* axis and that perpendicular to it. This magnetic anisotropy has been observed experimentally in the magnetic susceptibility measurements for a period of time^7^.

Moreover, from Fig. 1(d), we can see that there exist four inequivalent types of 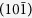 planes in which the spin chains are arranged in different ways, namely, the planes I to IV shown in Fig. 1(d). In I and III planes, the AHAFCs stretch along [111] direction [from left top to right bottom, see Fig. 1(c)]; while in planes II and IV, the chains go from left bottom to right top (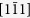 direction). The parallel chains in I(II) planes have a shift of 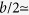 2.45 Å along *b* axis to those in nearest III(IV) planes as shown in Fig. 1(c).

### Electron density distributions of CN

We scraped together quite a number of experimental observations^7^,^8^,^23^,^25^ in the previous section, arriving at an AHAFC model description of CN. However, a thorough study of electronic structures in CN via *ab initio* calculations is indispensable, which may provide a direct check for the existence of spin-chain type magnetic interactions in CN and offers insight into exchange paths other than intra-chain couplings.

**Figure 2:**
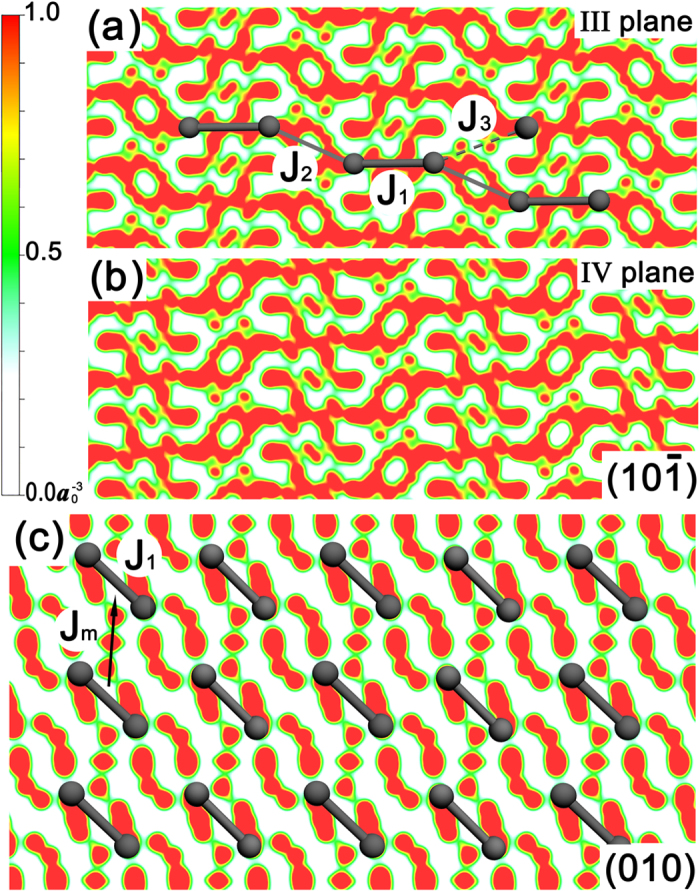
The electron density distributions. The projected electron densities on (**a**) III-type (10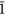), (**b**) IV-type (10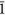), and (**c**) (010) planes. 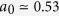 Å is the Bohr radius, the projection range of electron density is of thickness [−0.5, 0.5] *d (d* is the interplane distance), respect to [10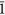] unit vector for (**a**,**b**) and to [010] vector (i.e., primitive vector *b*) for (**c**) (refer to Fig. 1 for the specific crystal directions). The positions of copper ions are marked by solid spheres. In (**a**,**b**) the tilted chain structures are clearly shown by high electron densities along the chain direction [111] for (**a**) and [1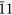] for (**b**). In (**c**) the dimers with different heights along *b* axis are labeled in different colors, from which it is clear that there exist weak inter-dimer interactions (denoted as *Jm*) along [001] direction, while there exists no visible exchange path between two nearest neighboring dimers connected by 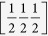 or 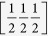 vector.

Figure 2 shows the simulated results of electron density distributions. Remarkably, in Fig. 2(a,b) the spin chain alignment in (10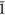) plane is clearly demonstrated, where the electrons tend to reside along the chain directions and thus leads to larger exchange integrals *J*_1_ and *J*_2_ [see Fig. 1(a)]. Note that from the calculated results, we can discriminate *J*_2_ from *J*_3_ without any ambiguity, where the Fig. 2 shows that the electron densities (hence also the coupling strengths) have different orders of magnitudes in *J*_2_ and *J*_3_ bonds. This conclusion, as well as the fact that the tilted chains are along difference directions between I, III and II, IV planes [Fig. 2(a,b)], agree with the INS observations in ref. 28. Moreover, in Fig. 2(c) we show the electron densities in (010) plane, where the *J*_1_ dimers are highlighted, from which we can see that there exist a weak dimer-dimer exchange coupling *J*_*m*_ between every pair of dimers along [001] direction, this again has been observed experimentally^23^.

In refs 23 and 26, INS experiments also show that there exists inter-chain interactions between nearest dimers along [001] and [100] directions. However, we find that by shifting dimers along [1/2 0 0] as indicated by the authors in refs 23 and 26, there locates *no* dimer in the supposed position (see Fig. 1). This is also verified in our *ab initio* calculations, where Fig. 2(c) shows clearly that there is no visible dimer-dimer coupling between a dimer and its nearest neighbor along [100] direction. Therefore, we include only the inter-dimer coupling *J*_*m*_ along [001] direction, and propose a novel 3D Heisenberg model (see in Supplementary Note 2), while leaving it as an open problem about the possibility of adding more inter-chain coupling terms to this 3D model [Eq. (S2)]. Note that the inter-chain interactions are rather weak and does not alter the physical properties except for ultra low temperatures. In the followings, the 3D model will not be involved, and we focus on the AHAFC model description in Eq. (1) exclusively.

### Thermal tensor network approach

High-precision thermal quantum manybody calculations are indispensable for relating the spin models discussed to the thermodynamical measurements of CN. The LTRG method, which is proposed by some of the authors, provides an accurate and efficient way to accomplish this task. Furthermore, we utilize here a double-layer algorithm LTRG++, which has significantly improved accuracy, compared to previous single-layer LTRG method, in computing thermodynamic properties (see some technical details in the Supplementary Note 3, and also a comprehensive discussion of LTRG++ for both spin and fermion models in ref. 56).

### Simulations of magnetothermal properties and precise coupling constants

We perform state-of-the-art TTN simulations developed and fit the magnetothermal data both taken from refs 7,8 and 22 and those measured in the present work. Various CN single-crystal specimens are prepared [see Supplementary Fig. S1] and we measure their magnetization in high-precision SQUID devices.

**Figure 3:**
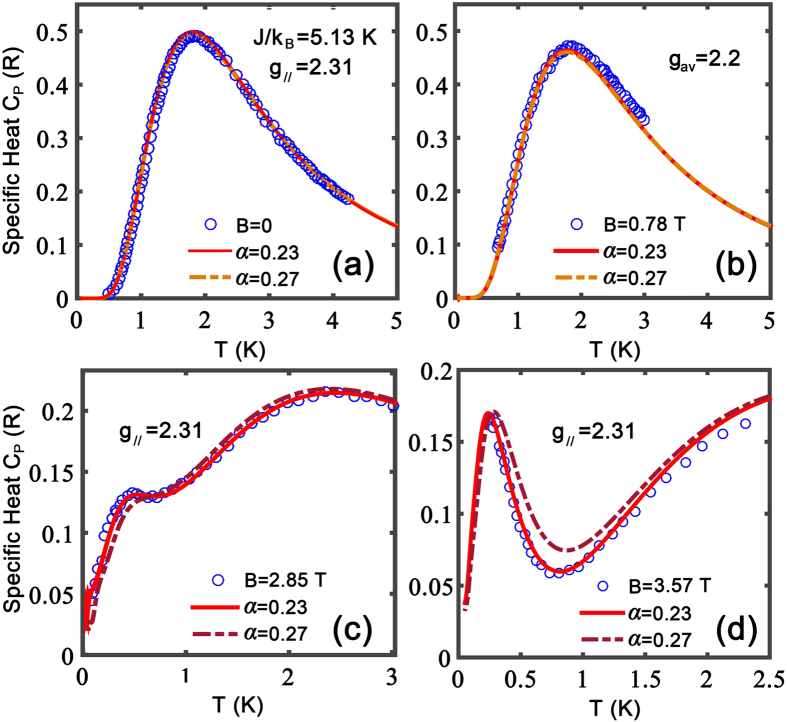
Fitting to experimental data of specific heat. The curves under various magnetic fields, (**a**) B = 0, (**b**) 0.78, (**c**) 2.85, and (**d**) 3.57 T. The experimental data (symbols) are taken from refs 8 and 22, and the dashed fitting lines are calculated with *α* = 0.27, while the solid lines are fittings with *α* = 0.23. The *B* = 0.78 T curve in (**b**) was measured with powder samples^22^, thus is fitted using average Landé factor 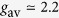; and the *B* = 2.82 and 3.57 T curves in (**c**,**d**) are measured along crystal *b* axis, with Landé factor 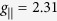.

We start from the specific heat curves at various magnetic fields *B* = 0, 0.87, 2.82, and 3.57 T, as shown in Fig. 3. Experimental data (symbols) are taken from ref. 22, and the coupling constant *J*, chosen to be 5.13 K, is within the fiducial range of 5.16(4) K from previous thermal fitting and 5.13(2) K from scattering fitting. Actually we find that small change of *J* (say, ±0.3 K) does not cause significant changes to calculated results so as to affect other fitting parameters.

In Fig. 3(a,b), we plot specific heat curves of low magnetic fields (*B* = 0, 0.78 T), and both fittings with *α* = 0.23 (solid lines) and 0.27 (broken lines) are displayed as comparisons. It is seen clearly that the calculated curves of both *α* values can fit the magnetic specific heat curves almost equally well, for either *B* = 0 or *B* = 0.78 T case. Therefore, it is difficult making a preferable choice amongst these two *α* values, as well as potentially many other values in between.

In Fig. 3(c) the measured specific heat curve *C*_*p*_ shows double peak structure, and *α *= 0.23 and 0.27 curves start to show some qualitatively different behaviors: While the *α* = 0.27 curve only presents a shoulder below 1 K, the *α* = 0.23 curve correctly captures the double-peak structure, making the latter fitting noticeably better than the former. Moreover, the difference between two fittings becomes more striking in Fig. 3(d), where *C*_*p*_ in the regime 0.3∼1.5 K is quite sensitive to the change of *α*, and *α* = 0.23 is obviously superior than 0.27 in this case.

Therefore, from the direct comparisons in Fig. 3, we conclude that *α* = 0.23 is an overall better parameter than *α* = 0.27 in the fittings of specific heat curves at various fields. The latter was obtained by the authors in ref. 22, who performed fittings based on ED results of small systems with the coupling ratio *α* = 0.27. We would like to stress that the discrimination between *α* = 0.23 and 0.27 can be done only if an accurately calculation is possible for the low-temperature thermodynamic property of CN at high fields (2.82 and 3.57 T) where the ground state is a critical Luttinger liquid.

**Figure 4:**
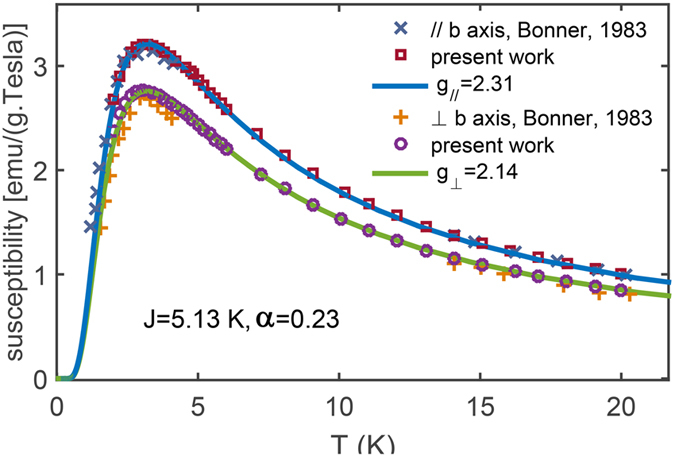
Fittings to measured magnetic susceptibility *χ*. The experimental data taken from previous experiments (ref. 22), as well as those obtained in the present work (squares and circles). The latter is measured under a small magnetic field (*B* = 0.6 T) to mimic the zero-field susceptibility. *χ* has clear anisotropic *g* factors along the crystal *b* axis and the direction perpendicular to it.

Then, we check whether the preferred parameter *α* = 0.23 can also fit other magnetothermal quantities such as zero-field susceptibility *χ* and the magnetization curves at various temperatures. Figure 4 illustrates the fittings to magnetic susceptibility results, which comprises data measured in the present work and those taken from ref. 22. In particular, the present magnetic susceptibility measurements are performed in order to fill up the gap in the temperature range 5 K < *T* < 15 K where the old susceptibility data are absent. It is seen that in Fig. 4 the TTN calculations can fit the experimental results very well. Note that the magnetic susceptibilities are measured both along and perpendicular to the crystal *b* axis, Fig. 4 reveals that there exists quite prominent anisotropy in the spin chain material. It turns out, through the fittings of both susceptibilities with Hamiltonian Eq. (1), that this anisotropy can be attributed to different Laudé factors in the directions parallel (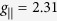) and perpendicular (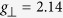) to the *b* axis.

**Figure 5:**
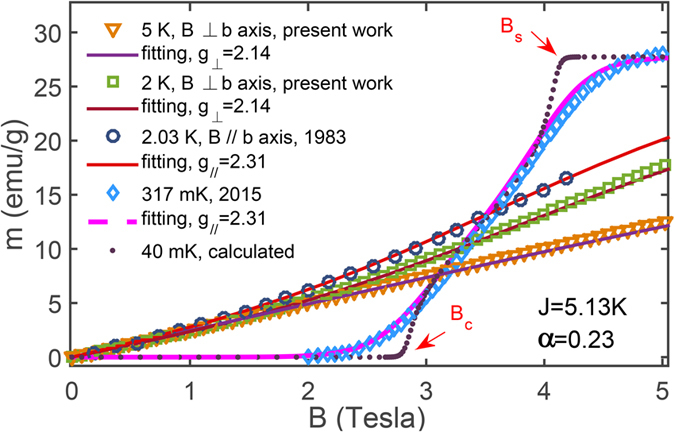
Various magnetization curves at different temperatures and their LTRG fittings. The two curves (at 2 and 5 K) under magnetic fields applied perpendicular to the *b* axis, are measured with a SQUID in the present work; while the 2.03 K curve parallel *b* axis and 317 mK curve perpendicular to *b* axis are taken from refs 22 and 29, respectively. A 40 mK line ideally calculated from the spin chain model is also included, demonstrating two quantum critical points *B*_*c*_ = 2.87 T and *B*_*s*_ = 4.08 T which are identified by two diverging peaks of *dM*/*dB*.

Besides the zero-field *χ*, we also fitted the magnetization curves at various temperatures (517 mK, 2, 2.03, and 5 K). In Fig. 5, the magnetization curves with fields perpendicular to *b* axis measured by us, parallel magnetization curves taken from ref. 22, as well as 517 mK data from ref. 29, are quantitatively fitted with the set of parameters *J* = 5.13 K, *α* = 0.23, 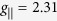, and 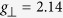.

### Criticality-enhanced magnetocaloric effect

In Fig. 5 the calculated magnetization at *T* = 40 mK, where two QCPs, i.e., the plateau-closing field *B*_*c*_ = 2.87 *T* and saturation field *B*_*s*_ = 4.08 T, are clearly shown. This ideal magnetization curve is calculated from 1D Hamiltonian Eq. (1) where inter-chain couplings are ignored. This curve is plotted just for elaborating two quantum critical points in the course of applying magnetic fields, and might have some distiction from realistic magnetization curve of CN since the interchain coupling might have some significant influence on the curve at such low temperatures (40 mK).

Between these two QCPs, there exists a continuous critical Luttinger liquid phase which hosts gapless magnetic excitations. The TTN simulations have be employed to explore the isentropes and magnetic Grüneisen parameters, and revisit the early isentropic data in ref. 20. We reveal that there exists criticality-enhanced MCE near two field-induced QCPs.

**Figure 6:**
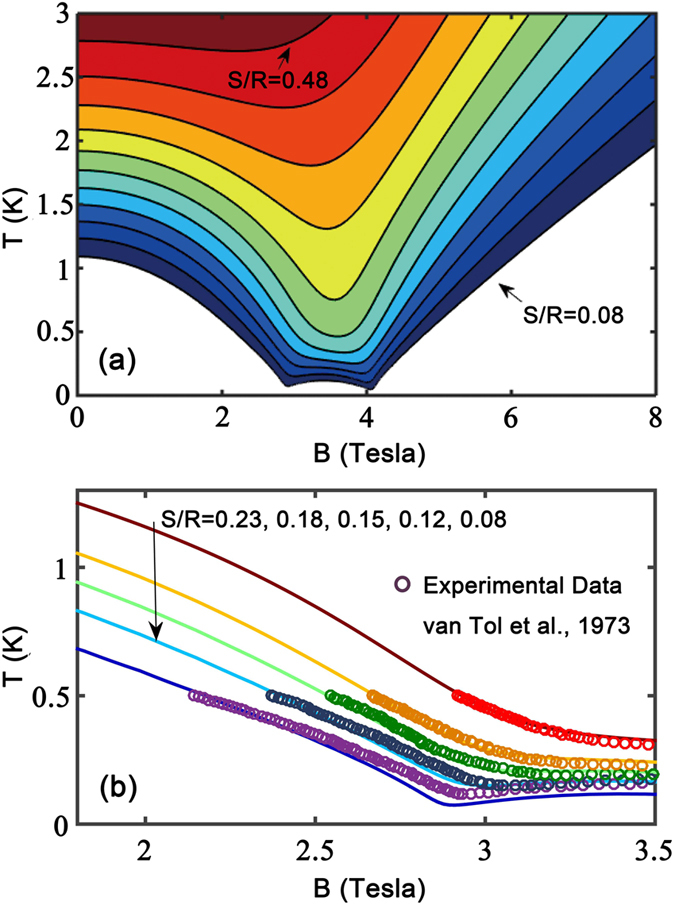
Numerically simulated and experimentally measured isentropes of CN. (**a**) The contour lines represent entropy per site 0.08 ≤ *S*/*R* ≤ 0.48 (bottom to top) with interval Δ*S*/*R* = 0.04, where 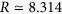 J·K^−1^·mol^−1^ is the gas constant. (**b**) Comparisons to measured adiabatic isentropes of CN around the critical field 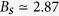 T, the experimental data are taken from refs 15 and 20.

In Fig. 6(a), we plot the isentropic curves of various magnetic entropies (from *S*/*R* = 0.05 to 0.5). For curves with relatively large entropies (0.2 ≤ *S*/*R*0.48), the lowest temperature appears at around 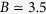 T, roughly located in the center of gapless region. However, with further lowering temperatures, we see that the broad dip eventually splits into two sharper dips in the isentropic curves, signalling two QCPs. Therefore Fig. 6(a) manifests that in the vicinities of QCPs and in the quantum critical region, the thermal entropies are relatively large, which in turn results in criticality-enhanced MCE.

Along each isentropic curve, one can read out the adiabatic temperature changes. A quite distinct future of Fig. 6(a) for CN chain is that on both small and large field sides, one experiences large temperature changes by varying fields (i.e. from 0 to 3 T, and 8 to 4 T). This is in contrast to uniform Heisenberg model (see, for instance, Fig. 3 in ref. 49 for spin chain material CuP), where significant MCE is observed only on large field (right) side of saturation QCP; while little temperature change was seen by increasing fields from 0 to saturation due to the presence of Luttinger liquid all along the magnetization curve. On the contrary, for the CN chain, the situation is different due to the existence of dimerization, which opens up a gap at low fields <*B*_*c*_. This fact enables us to realize criticality-enhanced MCE for relatively small fields (<4 T), and one could even properly design a thermal cycling to make use enhance MCE around both low and high critical fields in one complete cooling process.

In Fig. 6(b), we show the experimental data of isentropes (low-field region) taken from refs 15 and 20 and compare it to the simulated curves. From Fig. 6(b), we can see that, for isentropes with relatively large entropies (say, *S*/*R* = 0.23,0.18,0.15), the fittings based on 1D model [Eq. (1)] are strikingly good; when the entropy decreases and the lowest temperature obtained in the adiabatic experiments reaches ∼100 mK [see *S*/*R* = 0.08 in Fig. 6(b)], slight deviation starts to show up in the vicinity of QCP (*B*_*c*_ = 2.87 T). Such deviation may be ascribed to inter-chain interactions [see Supplementary Eqs. (S1,S2)] since the magnitudes of *J*_3_ and *J*_*m*_ are both about 0.01 meV (∼100 mK). Nevertheless, the good agreements to adiabatic temperature changes evidences that CN indeed has criticality-enhanced MCE characterized by large temperature change even for moderate fields (say, from 0 to 3.5 T).

**Figure 7:**
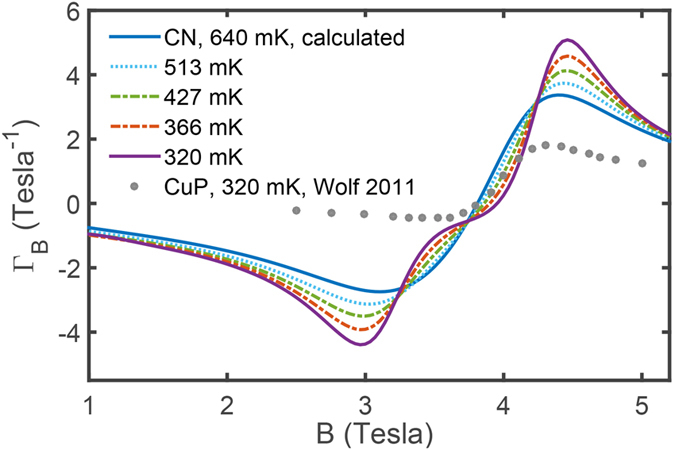
Magnetic Grüneisen parameter Γ_*B*_, which characterizes differentially the temperature change over a unit magnetic field change. The lines plotted, with different heights of peaks, correspond to different Γ_*B*_ at various temperatures, which decrease from 640 mK to 320 mK (top to bottom). The dotted line is the measured Γ_*B*_ of spin-1/2 Heisenberg chain model material CuP, taken from ref. 49.

**Figure 8:**
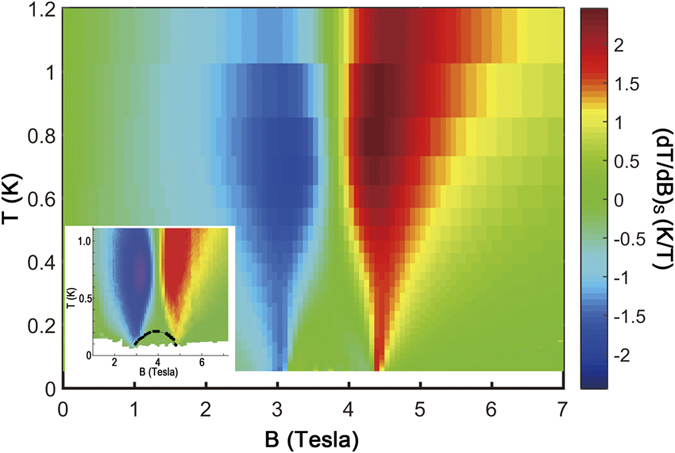
The calculated (*dT*/*dB*)_*s*_ versus magnetic fields and temperature, where the values of (*dT*/*dB*)_*s*_ are illustrated with colors. Inset is taken from ref. 58, which is obtained from experimental measurements. Note that the calculated color map bears remarkably similarity as the measured one.

Another important quantity measuring MCE property is the magnetic Grüneisen parameter 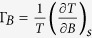, which is a differential characterization on the temperature change Δ*T* over small magnetic field variation Δ*B* in an adiabatic process. In the vicinity of QCPs, Γ_*B*_ diverges as *T* tends to zero, whose scaling behavior is intimately related to the quantum criticality^43^,^44^. In Fig. 7(a), we show the calculated Γ_*B*_ of CN, and also the measured Γ_*B*_ of uniform spin-1/2 Heisenberg chain material CuP as a comparison (taken from ref. 49), from which it is seen that the CN chain has much larger Γ_*B*_ around either one of its two QCPs, 2∼3 times as large as that of CuP around the saturation field. The latter has been proposed as a perfect alternative for ordinary demagnetization refrigerant due to its wide operating range, large cooling power, and high efficiency^49^. Our TTN simulations show that the dimerized spin chain CN studied in the present work has even more promising potential as quantum critical coolant, not only because it has two sharp dips at suitable fields (Fig. 6), one at *B*_*c*_ = 2.87 T and the other at *B*_*s*_ = 4.08 T, but also due to large temperature changes in response to field variations as revealed by ΓB in Fig. 7(a). In addition, we show in a color map the (*dT*/*dB*)_*s*_ as a function of various temperatures *T* and magnetic fields *B*, which turns out to resemble the experimental results with a high degree of similarity (inset in Fig. 8).

## Discussion

In this paper, we generalize the LTRG method to a bilayer form and employ this method to accurately study the thermodynamics of a 1D dimerized spin chain material copper nitrate. We calculate and fit the experimental data of specific heat, magnetic susceptibility, and magnetization curves, some of which are measured experimentally in the present work.

Through the large scale TTN simulations, we resolve the previous discrepancy in coupling constants verified from different experiments. In particular, since at strong field (2.8 T < B< 4.4 T) the ground state of the system is in a quantum critical regime (Luttinger liquid phase^29^) and is thus supposed to have rather long correlation lengths at low temperatures. Through high-precision fittings, we find that the verified coupling ratio *α* is close to that (*α* = 0.24) obtained from INS experiments^23^, while “measurably” different from *α* = 0.237 in previous papers^20^,^22^. The similar values *α* = 0.235 has been obtained by the ED fittings^29^. But due to finite-size effects, ED method is insufficient to give an accurate estimation of thermodynamic properties at low temperatures (like specific heat curves under critical fields). In contrary, Our TTN methods could directly access infinite-size chain and provide a faithful thermodynamic fitting.

Therefore, we conclude that the set of parameters *J* = 5.13 K, *α* = 0.23(1), 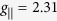, and 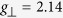 yielded from thermal fittings, is actually in remarkable consistency with those determined from INS experiments. This finding reveals that the thermal and scattering experiments are actually *consistent* with each other, and the previously supposed discrepancy may be due to limited simulations in fitting low-T thermal data of gapless Luttinger liquid phase. In addition, based on electron density distribution pattern, we have for the first time visualized the spin-chain exchange path in CN, through *ab initio* calculations.

Moreover, we uncover, though accurate TTN simulations of the model determined by thermal fittings, that there exists criticality-enhanced large MCE near two quantum phase transition points, even at very low temperatures. Based on the quantum anomaly in low-*T* isentropes and their good agreements to experimental data, as well as the large peaks/dips in magnetic Grüneisen parameters, we propose that CN is a very promising quantum critical coolant with significant temperature changes in response to magnetic field variations of moderate values.

There are still a number of interesting questions deserving further discussions, on both experimental and theoretical sides. To name a few, the direct experimental measurement of adiabatic temperature change for wider field ranges, instead of the rather limited field range between 2 to 4 T in previous experiments, is important to verify our prediction of CN as a promising coolant. In addition, the performance characteristics such as operation temperature range, cooling power, and efficiency, are also in due to be investigated. Another important ingredient missing in the present work is the effect of inter-chain couplings, as shown in the Supplementary Eqs (S1,S2). The inter-chain couplings could be of importance since the coolant is supposed to work in a circumstance with lowest temperature *T* < 100 mK, an energy scale comparable to inter-chain interactions.

## Methods

### Thermal quantum manybody computation

In order to simulate the thermodynamic properties and fit the experimental data, we employ thermal tensor network method to perform a high-precision calculation. In practices, Trotter slice is set as *τ* = 0.025, the lowest temperature reached is *T*/*J* = 1/150 (i.e., inverse temperature *β* = 150), and *χ* = 400∼600 bond states are retained, with truncation error smaller than 10^−13^. The numerical convergence versus *χ* of various concerned quantities including free energy, specific heat, magnetization curve, etc, has always been checked.

### First-principles calculations and electron density distributions

We employ a self-consistent field calculation, based on the all-electron projector augmented wave (PAW) method^59^,^60^ implemented in VASP^61^,^62^, to investigate the electron density distributions in CN. We adopt the generalized gradient approximation of Perdew, Burke, and Ernzerhof of exchange-correlation functional^63^. The cutoff energy for the plane wave expansion is chosen as 1000 eV, and the k-point mesh is 2 × 3 × 2. In practical calculations, little changes both in the cell shape and atomic positions have been observed after structure relaxation, hence the experimental lattice parameters shown in Fig. 2(a) are used, and two unit cells which comprise 264 atoms (including 16 copper atoms) are selected.

### Sample preparation and magnetization measurement

The Cu(NO_3_)_2_·2.5 H_2_O single crystals are obtained by cooling the hot saturated water solution of copper nitrate (CN) down to low temperatures. The solution was heated to increase the concentration, but the highest temperature should be below 75 °C to prevent copper nitrate from decomposition^8^. In practice, we heat the hot solutions to 75 °C, and then transfer it directly to a cooler container (<25 °C) to facilitate crystal seed formation. The temperature of latter controls the final single-crystal size of the specimen. Sequentially, the container is put into the 60 °C environment, which is slowly cooled down to 35 °C. The grown single crystals have quite large system sizes, ranging from several milimeters to one or two centimeters (see Supplementary Fig. S1), and are in needle shapes with the long edge right along the crystal *b* axis. Note during the process of sample preparation, the CN solution should be kept away from organic materials or solution.

We point out that there exist more than one kind of copper nitrate hydrates. In order to ensure the purity of Cu(NO_3_)_2_·2.5 H_2_O in the specimen (i.e., to remove superfluous water and other possible CN hydrates), the sample is heat to 45 °C for 10 min everytime before measurements^27^. The data in Figs 4 and 5, including the isothermal magnetization curves and the zero-field susceptibility are measured in the high-precision SQUID device by scanning fields and temperatures, respectively.

## Additional Information

**How to cite this article:** Xiang, J.-S. *et al*. Criticality-Enhanced Magnetocaloric Effect in Quantum Spin Chain Material Copper Nitrate. *Sci. Rep.* **7**, 44643; doi: 10.1038/srep44643 (2017).

**Publisher's note:** Springer Nature remains neutral with regard to jurisdictional claims in published maps and institutional affiliations.

## Supplementary Material

Supplementary Information
