# Supplementary material for: Criticality-Enhanced Magnetocaloric Effect in Quantum Spin Chain Material Copper Nitrate
Source: Sci Rep. 2017 Mar 15;7:44643. doi: 10.1038/srep44643 (PMC5353727; doi:10.1038/srep44643)
Supplement: Supplementary Information [file srep44643-s1.pdf]

SUPPLEMENTARY INFORMATION

Criticality-Enhanced Magnetocaloric Effect in Quantum Spin Chain Material Copper Nitrate

Jun-Sen Xiang,[1] Cong Chen,[1] Wei Li,[1,2,*] Xian-Lei Sheng,[1,3] Na Su,[4] Zhao-Hua Cheng,[4] Qiang Chen,[1] and Ziyu Chen[1,†]

[1]*Department of Physics, Key Laboratory of Micro-Nano Measurement-Manipulation and Physics (Ministry of Education), Beihang University, Beijing 100191, China*
[2]*International Research Institute of Multidisciplinary Science, Beihang University, Beijing 100191, China*
[3]*Department of Physics and Astronomy, University of Delaware, Newark, Delaware 19716-2570, USA*
[4]*State Key Laboratory of Magnetism and Beijing National Laboratory for Condensed Matter Physics, Institute of Physics, Chinese Academy of Sciences, Beijing 100190, China*

* w.li@buaa.edu.cn
† chenzy@buaa.edu.cn

SUPPLEMENTARY FIGURE

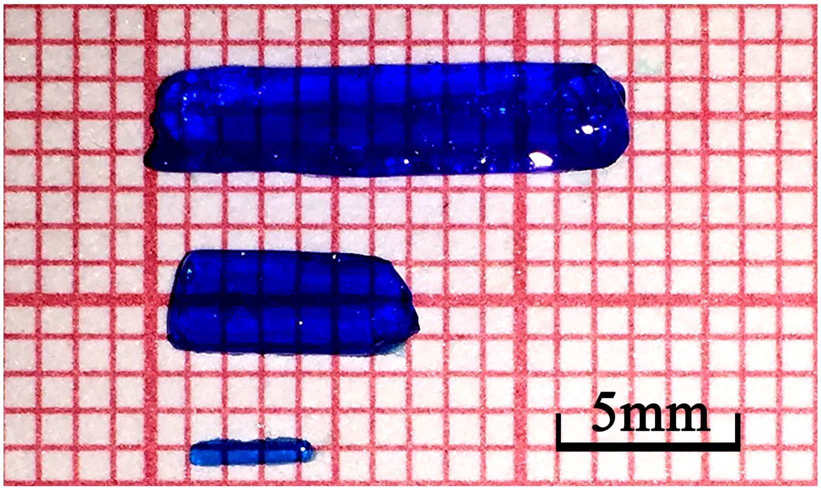

Supplementary Figure S 1. Examples of synthesized single-crystal CN specimens, which are transparent and with needle-like shapes. The needle axis is parallel to crystal *b* axis, and the lengths of samples range from a few mm to more than 1 cm.

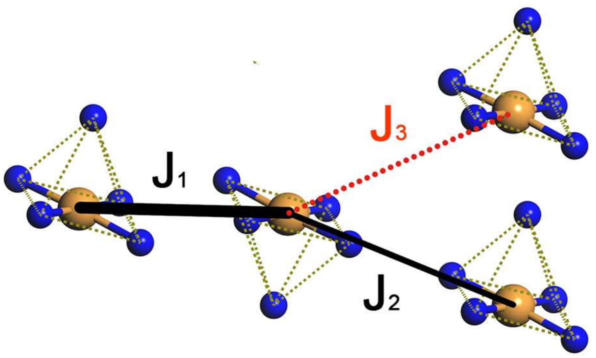

Supplementary Figure S 2. The expanded view of the quare pyramidal polyhedrons comprise of oxygen atoms and Cu^{2+} on the $(10\bar{1})$ plane. Each Cu^{2+} is surrounded by five nearest-neighboring oxygen atoms, which constitutes a distorted pyramidal polyhedron. Four oxygen atoms reside at the vertices of the basal plaquette of the polyhedron, roughly on the same plane: two of these oxygen atoms belong to H_2O molecules and the other two are from NO_3^- groups; the rest apical oxygen atom belongs to a third NO_3^- group. The pyramidal polyhedrons of opposite orientations are arranged alternatively along a line.

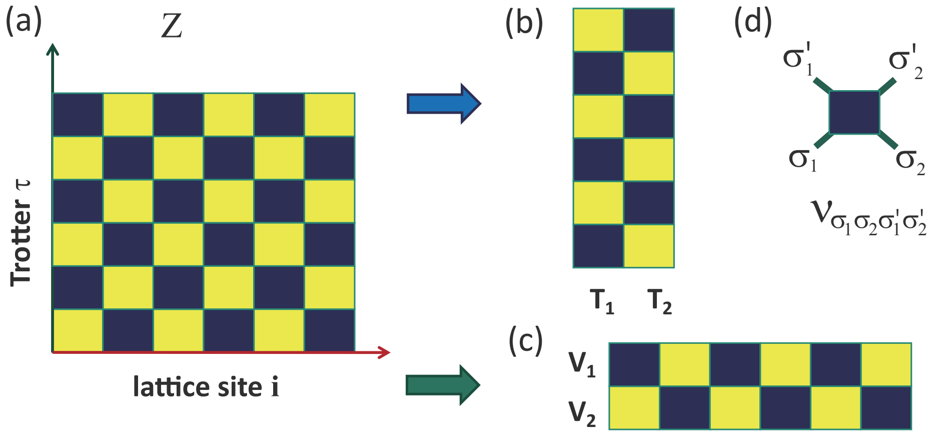

Supplementary Figure S 3. (**a**) The 2D TTN represents the partition function **Z** of a 1D quantum lattice model, which exhibits a checkerboard pattern. (**b**) and (**c**) are the transfer matrices along spatial and Trotter directions, respectively. (**d**) depicts the rank-four local tensor ν, the elementary unit in the TTN. Note here that during the process of contracting thermal tensor networks, the tensor elements in MPO becomes larger and larger as temperature continues to cool down, and eventually diverges. Therefore, after each single substep of projecting ν tensors to MPO, one need to extract some renormalization factor κ from the MPO to avoid divergence, see more details in Ref. 1

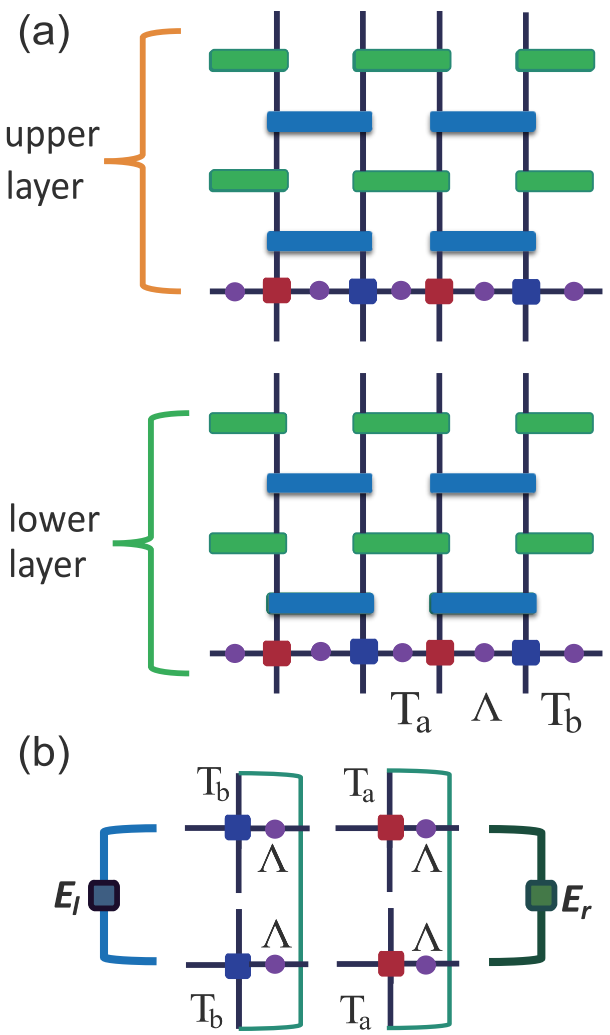

Supplementary Figure S 4. **LTRG++ algorithm** (**a**) adopts a double-layer scheme which contracts simultaneously upper and lower layers into two MPOs. (**b**) $T_{a(b)}$ and its conjugate counterpart constitute the transfer matrix, with the left(right) dominant eigenvector $E_{l(r)}$ and the corresponding eigenvalue $\lambda_{\max}$.

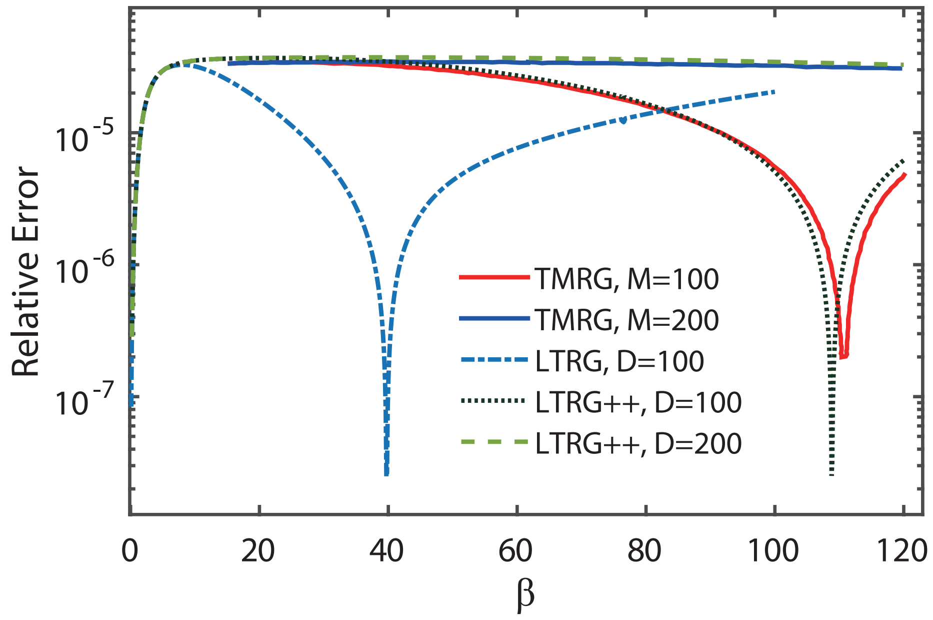

Supplementary Figure S 5. Relative errors of free energy per site in LTRG, LTRG++, and TMRG, for the exactly soluble model 1D free fermion chain. M and D are the number of retained states during the process of projection and truncation in LTRG/LTRG++ and TMRG, respectively.

SUPPLEMENTARY NOTE

Supplementary Note 1. The Review of Copper Nitrate

We should note that there exist more than one kind of copper nitrate hydrates. Among others, the $Cu(NO_3)_2 \cdot 3H_2O$ (copper nitrate trihydrate) has similar formulas and exactly the same X-ray diffraction pattern [2], and thus is easy to be confused with CN hemipentahydrate studied here. We paid special attention to discriminate between these two similar hydrates, and according to thermogravimetric analysis [3, 4], it is concluded that $Cu(NO_3)_2 \cdot 2.5H_2O$ is a distinct hydrate different from $Cu(NO_3)_2 \cdot 3H_2O$.

Based on early experimental research on CN, including the measurements of magnetic susceptibility [5] and specific heat [6], a binary cluster model for describing its magnetic properties was proposed: Cu^{2+} having spin $S = 1/2$ are coupled in pair (with coupling strength $\frac{1}{2}J/k_B = 2.56$ K) [5], and the system thus comprises of independent spin binary clusters. In addition, people also perform proton magnetic resonance (PMR) experiments and confirm that the short-range antiferromagnetic order are within spin-pairs, thus supporting the dimer model. [7]

Although the binary cluster model can capture some of the main features of spin-spin correlation in CN, discrepancy between this simple model and experimental measurements still remains. A weak interdimer exchange interaction (J_2) was then brought into the binary cluster model in Ref. 8, which substantially improves the fitting to the isothermal magnetization curve. In addition, van Tol, *et al.*, studied the magnetic phase transitions via PMR and uncovered a crossover to short-range magnetic order at 350 mK, as well as a critical transition to long-range order at 160 mK, under a magnetic field of 3.6 T, which can be understood only by introducing inter-dimer couplings in the model [9]. Besides the PMR data, this is also evidenced in the specific heat measurement, where the low temperature hump(peak) signals the crossover(transition) into short-(long-) range ordered state [10–12], also suggesting the existence of weak inter-dimer couplings.

Early attempts to introduce inter-dimer interactions include two possible model structures: a ladder model (with dimers on the rungs) and an alternating chain model [13]. These two models both fit thermodynamic measurements of CN well since their corresponding thermal predictions are essentially equal [12, 14]. However, the discrimination between these two models was later done by the angular dependent PMR [15], according to which one identifies the possible superexchange paths and thus rules out the ladder model. Since 1980s, the community has achieved an agreement that the microscopic model of CN is very well approximated by a dimerized spin chain (above the AF transition temperature). Even though, CN continues to be an intriguing model material and attracts a lot of research interest. For example, people has synthesized more copper(II)-based complexes [16–19], and focused on its exotic thermal properties around quantum critical points [20–22]. In addition, inelastic neutron scattering, as a powerful tool investigating magnetic properties, has been applied to probe the microscopic magnetic structures, magnetic ordering and excitation dispersion of CN [23–25].

Supplementary Note 2. Novel 3D Heisenberg spin model

The AHAFC model can explain well most experimental observations of CN in a very wide range of temperatures (above a few hundreds of milikelvins). Nevertheless, ultra low temperature measurements of PMR [9], adiabatic susceptibility [11], and heat capacity [14], uncovered a 3D phase transition at about $150 \sim 160$ mK, under magnetic fields between B_c and B_s. The existence of such a finite-temperature transition from magnetic disordered phase to 3D long-range ordered phase [21] is clearly beyond the 1D spin model given in Eq. (1)).

We mentioned that the ferromagnetic coupling J_3 whose magnitude is about 1/10 of J_2 [Fig. S2, Fig. 1(b)], and thus plays the role of a weak inter-chain interaction. In addition, INS experiments reveal that there exist some other inter-chain interactions, namely, $J_m = 0.018(2)$ meV, between dimers and along [001] directions [Fig. 1(d)] [24]. These important facts are confirmed by our first-principles calculations, as Fig. 2 illustrates.

Put all these intra- and inter-chain interactions together, we arrive at a 3D Heisenberg spin model with Hamiltonian $H_{3D} = H_{(10\bar{1})} + H_{\rm CTC}$, which reads:

$$H_{(10\bar{1})} = \sum_{\vec{p}\in\{A\}} J_1 \vec{S}_{\vec{p}} \vec{S}_{\vec{p}+\hat{i}} + J_j \vec{S}_{\vec{p}} \vec{S}_{\vec{p}+\hat{j}} + J_k \vec{S}_{\vec{p}} \vec{S}_{\vec{p}+\hat{k}}, \tag{S1}$$

and

$$H_{\rm CTC} = \sum_{\vec{p}} J_m \vec{S}_{\vec{p}} \vec{S}_{\vec{p}+\vec{c}/2}. \tag{S2}$$

Equation (S1) is a 2D honeycomb lattice model with three different coupling constants along $\hat{\imath}$, $\hat{\jmath}$, and $\hat{k}$ directions [see Fig. 1(d)]. $\vec{p}$ is the coordination vector in $\{A\}$ sublattice [Eq. S1] or running over all lattice sites [Eq. S2] of honeycomb lattice on $(10\bar{1})$ planes. One typical site in $\{A\}$ sublattice has been marked in Fig. 1(d). Note that $J_j = J_2$ and $J_k = J_3$ on planes I and III, while $J_k = J_2$ and $J_j = J_3$ on planes II and IV.

Equation (S2) represents the inter-layer couplings, J_m is the dimer coupling, $\vec{p}$ is coordination vector of every localized spin site, and $\vec{c}$ has magnitude of lattice constant $c = 15.8$ Å and is along [001] direction (there must be a spin site at $\vec{p} + \vec{c}/2$ according to CN structure shown in Fig. 1). Some additional remarks on 3D inter-chain couplings in Eq. (S2) are in order: Since there exist four kinds of 2D honeycomb planes [I to IV in Figs. 1(c,d)] which are different from each other by lattice shifts (and reflection) operations about (010) plane, and the chains are arranged along different paths ([111] or $[11\bar{1}]$) on different planes, Eq. S2 thus describes a 3D coupled tilted chains (CTC) model. In this model, the dimers are coupled via J_m when they are separated by $c/2$ along [001] direction in ac-plane. Therefore, the seemingly simple H_{CTC} in Eq. (S2) actually represents a quite peculiar 3D inter-chain coupling model which looks bizarre while are actually feasible in the material CN. This 3D model has not been reported before as far as we know, neither has its properties been explored.

Supplementary Note 3. Thermal Tensor Networks and Linearized Tensor Renormalization Group Method

Employing the Trotter-Suzuki decomposition [26], we obtain a 2D Thermal Tensor Networks (TTN) for the 1D Heisenberg chain, which consists of rank-four tensors $\nu_{\sigma_1,\sigma_2,\sigma_3,\sigma_4} = \langle\sigma_1,\sigma_2|\exp(-\tau h_{i,j})|\sigma_3,\sigma_4\rangle$ [see Fig. S3 (d)]. In order to calculate the thermodynamic properties, one needs to accurately contract the TTN, which, however, is a NP-hard problem and thus can not be solved exactly. Therefore, people has to resort to approximate methods for efficient contractions of TTN. Among others, renormalization group (RG) algorithms constitute an important class of approaches which are developed to accurately contract the TTN and calculate interested quantities including free energy per site, specific heat, magnetic susceptibility, entropy, and others.

As shown in Fig. S3, the interconnected local tensors constitute a 2D checkerboard-style TTN, which subsequently can be regarded as repeated 1D vertical (T_1, T_2) or horizontal (V_1, V_2) stripes, i.e., transfer matrices, as shown in Figs. S3 (b,c). The full contraction of TTN and consequently the calculations of thermal properties can be accomplished with the help of these transfer matrices. For instance, the vertical stripes T_1, T_2 transfers the spin indices $\{\sigma_i\}$ between spatially different lattice sites and the partition function Z thus reads $Z = \lim_{L\to\infty} \mathrm{Tr}(\mathrm{T_1 T_2})^{\mathrm{L}/2}$, where L denotes the total length of the chain. In the thermodynamic limit the dominant eigenvalue $\lambda_{\max}$ of transfer matrix $T = T_1T_2$ determines the free energy per site $f = \frac{1}{2\beta}\ln\lambda_{\max}$ and also other thermodynamic quantities.

In order to calculate the extreme eigenvalue (and corresponding eigenvector), in Ref. 27–29, Xiang and Wang utilized the density-matrix renormalization group (DMRG) method [30, 31], originally developed for Hamiltonian system, to solve the transfer matrix problem. They perform RG process along the Trotter direction and truncate the accumulated $\{\sigma_i\}$ indices/states into a fixed number (M) of renormalized states. Transfer matrix renormalization group (TMRG) can determine the thermodynamic properties with high precision, and has been established as the method of reference for 1D quantum lattice systems [32–34].

Alternatively, efficient contractions of the 1+1D TTN can also be performed by making use of the horizontal transfer matrices V_1, V_2 [see Fig. S3 (c)]. Li *et al.* proposed a TTN algorithm dubbed as LTRG [1], which projects continually the transfer matrix $V_{1(2)}$ to the density matrix of the system (in a form of matrix product operator, MPO). The linearized tensor renormalization group (LTRG) method can be used to accurately calculate the thermodynamics in 1D chains [1, 35] and applies also to higher dimensional lattices [1, 36].

Besides the single-layer algorithm, we devise here a double-layer LTRG++ algorithm, as shown in Fig. S4, for contracting the TTN. The main idea is as following: we contract the TTN into *two* (instead of one) MPOs (this is what "++" means). Figure S4(a) exploits a symmetric construction, where the upper and lower layers are contracted into two MPOs in exactly the same manner, saving one half of the projection time. Due to the checkerboard structure of TTN, each projection step comprises of two substeps, called the odd and even substeps. After n steps, one reaches the inverse temperature $\beta = (2n+2)\tau$. The free energy per site $f(\beta) = \lim_{N\to\infty}[-\frac{1}{N}\log(Z)]$ can now be calculated from the series of renormalization factors κ^a and κ^b, extracted at odd and even substeps, respectively. In addition, we also need to calculate the dominant eigenvalue $\lambda_{\max}$ of transfer matrix consisted of T_a, T_b and their conjugates in Fig. S4(b).

In Fig. S5 we compare numerically the accuracy of single-layer LTRG, TMRG and bilayer LTRG++ results for the exactly soluble XY spin chain model with Hamiltonian $H_{\mathrm{XY}} = \sum_i S_i^x S_{i+1}^x + S_i^y S_{i+1}^y$. There are two kinds of error in these methods: for small β (high temperatures regime) Trotter error is dominant, while for large β (low temperature regime) truncation error takes the place. Note that two errors take opposite signs so they cancel each other at the cancelation point β_s. The position of β_s is controlled by the bond dimension D, and also heavily related to the specific truncation scheme adopted. Fig. S5 depicts that when the number of bond states $D = 100$, LTRG++ and TMRG have almost the same β_s, much larger than that of LTRG; and when D is as large as 200, β_s locates further than 120 in both LTRG++ and TMRG. When the cancelation point β_s moves further along the horizontal axis, the accuracy of simulations improves generally (note that the total errors, mainly contributed

by truncations, grow up very fast after the cancelation point, i.e, $\beta > \beta_s$).

Therefore, as seen from the Fig. S5, bilayer LTRG++ has significant improvement compared to the single-layer LTRG, and bears practically the same accuracy as TMRG. This observation strongly supports our argument above that LTRG++ is in essence equivalent to TMRG.

In conclusion, the advantages of LTRG++ can be summarized in mainly two aspects: Firstly, LTRG++ further improves the accuracy (compared to LTRG), it is now practically of the same precision as TMRG, and can even save half of the projection time. Secondly, TTN approaches are very *flexible*, and can be designed to rewrite/recover the well-established TMRG, purification, and potentially other thermal RG methods. For instance, one can adopt a symmetric construction in LTRG++ such that two MPOs are (approximately) conjugate to each other, and recovers then the purification scheme [37] used in finite-temperature DMRG [38]. Moreover, the success of LTRG++ in 1D also encourages us to generalize it to higher dimensions. See more technical details and systematic discussions on LTRG++ in Y.-L. Dong, et al., arXiv:1612.01896 (2016).

SUPPLEMENTARY REFERENCES

[1] W. Li, S. J. Ran, S. S. Gong, Y. Zhao, B. Xi, F. Ye, and G. Su, *Linearized Tensor Renormalization Group Algorithm for the Calculation of Thermodynamic Properties of Quantum Lattice Models*, Phys. Rev. Lett. **106**, 127202 (2011).

[2] F. A. H. Schreinemakers, G. Berkhoff, and K. Posthumus, *Le Système: Cu(NO$_3$)$_2$-NH$_4$NO$_3$-H$_2$O*, Recueil des Travaux Chimiques des Pays-Bas **43**, 508-511 (1924).

[3] T. J. Taylor, D. Dollimore and G. A. Gamlen, *Deaquation and denitration studies on copper nitrate trihydrate*, Thermochimica. Acta. **103**, 333-340 (1986).

[4] I. V. Morozov, K. O. Znamenkov, Yu. M. Korenev, O. A. Shlyakhtin, Thermal decomposition of $Cu(NO_3)_2 \cdot 3H_2O$ at reduced pressures, Thermochimica. Acta. **403**, 173-179 (2003).

[5] L. Berger, S. A. Friedberg, and J. T. Chriempf, *Magnetic Susceptibility of $Cu(NO_3)_2 \cdot 2.5H_2O$ at Low Temperature*, Phys. Rev. **132**, 1057-1061 (1963).

[6] S. A. Friedberg and C. A. Raquet, *The Heat Capacity of $Cu(NO_3)_2 \cdot 2.5H_2O$ at Low Temperatures*, Journal of Applied Physics **39**, 1132 (1968).

[7] S. Wittekoek and N. J. Poulis, *Proton Magnetic Resonance Study of Magnetic Ordering in Two Cupric Salts*, Journal of Applied Physics **39**, 1017 (1968).

[8] B. E. Myers, L. Berger, and S. A. Friedberg, *Low-Temperature Magnetization of $Cu(NO_3)_2 \cdot 2.5H_2O$*, J. Appl. Phys. **40**, 1149 (1969).

[9] M. W. van Tol, L. S. J. M. Henkens, and N. J. Poulis, *High-Field Magnetic Phase Transition in $Cu(NO_3)_2 \cdot 2.5H_2O$*, Phys. Rev. Lett. **27**, 739 (1971).

[10] M. W. Van Tol, J. P. Groen and N. J. Poulis, *Specific heat and NMR of $Cu(NO_3)_2 \cdot 2.5H_2O$ at the high-field phase transition*, Physica **64**, 363 (1973).

[11] K. M. Diederix, J. P. Groen and N. J. Poulis, *A study of the high field phase transition in $Cu(NO_3)_2 \cdot 2.5H_2O$*, Physica **86**, 1151 (1977).

[12] K. M. Diederix, et al., *An experimental study on the magnetic properties of the singlet ground-state system in $Cu(NO_3)_2 \cdot 2.5H_2O$: I. Short-range ordered state*, Physica **93B**, 99 (1978).

[13] M. Tachiki and T. Yamada, *Spin Ordering and Thermodynamical Properties in Spin-Pair Systems under Magnetic Fields*, Prog.Theor.Phys.Suppl. **24**, 291 (1970).

[14] J. C. Bonner, S.A. Friedberg, H. Kobayashi, D.L. Meier, H.W.J Blote, *Alternating linear-chain antiferromagnetism in copper nitrate $Cu(NO_3)_2 \cdot 2.5H_2O$*, Phys. Rev. B. **27**, 248 (1983).

[15] K. M. Diederix, et al., *An experimental study on the magnetic properties of the singlet ground-state system in $Cu(NO_3)_2 \cdot 2.5H_2O$: II. The long-range ordered state*, Physica **94B**, 9 (1978).

[16] J. Li, J. Zhang, Y. Ke, and X. Wu, *A Novel Structure of Bipyridine Coordinated with Copper (II): $[Cu(bipy)(H_2O)_3] \cdot (NO_3)_2$*, Crystal Research & Technology **31**, 589 (1996).

[17] Takayuki Ishida, et al., *Magnetic properties of pyrimidine-bridged copper(II) complexes,* Synthetic metals **85**, 1655 (1997).

[18] A. M. Thomas, et al., *Synthesis, crystal structure, and nuclease activity of planar mono-heterocyclic base copper(II) complexes,* Journal of Inorganic Biochemistry **94**, 171 (2003).

[19] E. V. Peresypkina, et al., *Bis(benzotriazol-1-yl)methane as a linker in the assembly of new copper(II) coordination polymers: Synthesis, structure and investigations,* Polyhedron **48**, 253 (2012).

[20] B. Wolf, et al., *Cooling through quantum criticality and many-body effects in condensed matter and cold gases*, International Journal of Modern Physics B **28**, 1430017 (2014).

[21] B. Willenberg, et al., *Luttinger liquid behavior in the alternating spin-chain system copper nitrate*, Phys. Rev. B. **91**, 060407(R) (2015).

[22] Gegenwart, P. *Grüneisen parameter studies on heavy fermion quantum criticality*, Rep. Prog. Phys. **79**, 114502 (2016).

[23] J. Eckert, D.E. Cox, G. Shirane, S.A. Friedberg, H. Kobayashi, *Magnetic ordering in $Cu(NO_3)_2 \cdot 2.5D_2O$,* Phys. Rev. B. **20**, 4596 (1979).

[24] Guangyong Xu, C. Broholm, Daniel H. Reich, and M. A. Adams, *Triplet Waves in a Quantum Spin Liquid*, Phys. Rev. Lett. **84**, 4465 (2000).

[25] D. A. Tennant, et al., *Anomalous dynamical line shapes in a quantum magnet at finite temperaturePhys*, Phys. Rev. B **85**, 014402 (2012).

[26] M. Suzuki, *Relationship between d-Dimensional Quantal Spin Systems and (d+1)-Dimensional Ising Systems: Equivalence, Critical*

Exponents and Systematic Approximants of the Partition Function and Spin Correlations, Prog. Theor. Phys. **56**, 1454 (1976).

[27] R.J. Bursill, T. Xiang and G.A. Gehring, *The density matrix renormalization group for a quantum spin chain at non-zero temperature*, J. Phys.: Condes. Matter **8**, L583 (1996).

[28] X. Wang and T. Xiang, *Transfer-matrix density-matrix renormalization-group theory for thermodynamics of one-dimensional quantum systems*, Phys. Rev. B **56**, 5061 (1997).

[29] T. Xiang, *Thermodynamics of quantum Heisenberg spin chain*, Phys. Rev. B **58** 9142 (1998).

[30] S. R. White, *Density Matrix Formulation for Quantum Renormalization Groups*, Phys. Rev. Lett. **69**, 2863 (1992).

[31] S. R. White, *Density-matrix algorithms for quantum renormalization groups*, Phys. Rev. B **48** 10345 (1993).

[32] X. Wang and L. Yu, *Magnetic-Field Effects on Two-Leg Heisenberg Antiferromagnetic Ladders: Thermodynamic Properties*, Phys. Rev. Lett. **84**, 5399 (2000).

[33] J. Lou, T. Xiang, and Z.B. Su, *Thermodynamics of the Bilinear-Biquadratic Spin-One Heisenberg Chain*, Phys. Rev. Lett. **85**, 2380 (2000).

[34] B. Gu and G. Su, *Comment on "Experimental Observation of the 1/3 Magnetization Plateau in the Diamond-Chain Compound* $Cu_3(CO_3)_2OH_2$*"*, Phys. Rev. Lett. **97**, 089701 (2006).

[35] X. Yan, W. Li, Y. Zhao, S.-J. Ran, and G. Su, *Phase diagrams, distinct conformal anomalies, and thermodynamics of spin-1 bond-alternating Heisenberg antiferromagnetic chain in magnetic fields*, Phys. Rev. B **85**, 134425 (2012).

[36] S.-J. Ran, W. Li, B. Xi, Z. Zhang, and G. Su, *Optimized decimation of tensor networks with super-orthogonalization for two-dimensional quantum lattice models*, Phys. Rev. B **86**, 134429 (2012).

[37] F. Verstraete, and J. I. Cirac, Renormalization algorithms for Quantum-Many Body Systems in two and higher dimensions, *cond-mat/0407066 (unpublished)*.

[38] A. E. Feiguin, and S. R. White, Finite-temperature density matrix renormalization using an enlarged Hilbert space, *Phys. Rev. B* **72**, 220401(R) (2005).
